# A high-content image-based method for quantitatively studying context-dependent cell population dynamics

**DOI:** 10.1038/srep29752

**Published:** 2016-07-25

**Authors:** Colleen M. Garvey, Erin Spiller, Danika Lindsay, Chun-Te Chiang, Nathan C. Choi, David B. Agus, Parag Mallick, Jasmine Foo, Shannon M. Mumenthaler

**Affiliations:** 1 Lawrence J. Ellison Institute for Transformative Medicine, University of Southern California, Los Angeles, California, USA; 2 School of Mathematics, University of Minnesota, Minneapolis, Minnesota, USA; 3 Department of Radiology, Canary Center at Stanford for Cancer Early Detection, Stanford University, Palo Alto, California, USA

## Abstract

Tumor progression results from a complex interplay between cellular heterogeneity, treatment response, microenvironment and heterocellular interactions. Existing approaches to characterize this interplay suffer from an inability to distinguish between multiple cell types, often lack environmental context, and are unable to perform multiplex phenotypic profiling of cell populations. Here we present a high-throughput platform for characterizing, with single-cell resolution, the dynamic phenotypic responses (i.e. morphology changes, proliferation, apoptosis) of heterogeneous cell populations both during standard growth and in response to multiple, co-occurring selective pressures. The speed of this platform enables a thorough investigation of the impacts of diverse selective pressures including genetic alterations, therapeutic interventions, heterocellular components and microenvironmental factors. The platform has been applied to both 2D and 3D culture systems and readily distinguishes between (1) cytotoxic versus cytostatic cellular responses; and (2) changes in morphological features over time and in response to perturbation. These important features can directly influence tumor evolution and clinical outcome. Our image-based approach provides a deeper insight into the cellular dynamics and heterogeneity of tumors (or other complex systems), with reduced reagents and time, offering advantages over traditional biological assays.

Tumors are dynamic, evolving systems whose multi-layered complexity stems from a number of microenvironmental features including oxygen and nutrient gradients and interactions among diverse cell types (host and tumor, different molecular subtypes)^1^,^2^,^3^. In preclinical studies, it has become increasingly important to capture the heterogeneity of tumors at the cellular and microenvironmental level in order to recapitulate a more realistic environmental context to study tumor progression and therapeutic response^4^,^5^,^6^. Ignoring contextual influences when exploring these areas of research can be misleading.

To determine cellular response to a perturbation, traditional biology assays for cell viability (e.g. MTS, alamar blue, Annexin V-FITC flow cytometry assay) are widely employed. Although these assays are robust and easy to perform, they often fail to provide a complete picture of cellular events^7^. In particular, they tend to overlook alterations in additional cellular phenotypes, are not amenable to co-culturing different cell types, and are prone to missing absolute changes in cell population composition. These assays frequently depend on surrogate measurements of cell number (e.g. ATP levels or DNA content) and the data is expressed as relative events rather than as absolute cell counts. One major limitation of cell viability readouts is that they do not reveal whether a cell population has undergone growth arrest, cell death, or a combination of both when exposed to a perturbation. For example, a readout of 50% cell viability after exposure to drug “x” can be interpreted as either (1) the cell doubling time was twice as long compared to the control population, or (2) twice as many cells died compared to the control population. Clinically, this has significant implications for predicting tumor growth kinetics, drug treatment response, and the likelihood of emergence of drug resistance, which translates to not being able to distinguish between drugs that are capable of inducing tumor shrinkage (cell death) versus prompting growth arrest (cell cycle arrest).

Tumor compositions are extremely heterogeneous and many traditional viability assays are unable to decouple the effects from multiple cell types or resolve the heterogeneity found within a single cell type/subpopulation.ySpecifically, many sources of cell-to-cell variation exist in tumors including different types of cells (e.g. fibroblasts versus epithelial cells) or the same ‘type’ of cell (e.g. epithelial) with several clones that have acquired different mutations. Flow cytometry is a commonly used technique that is able to differentiate between cellular populations when fluorescently labeled and can analyze a large number of cells in a short period of time. However, cell labels are a prerequisite, dynamic observations are not possible, and, for adherent cells, the assay preparation is time consuming and error-prone (e.g. loss of live and dead cells during washes). Further, cell morphology information, which has recently been shown to correlate with tumor subtypes^8^ and aggressivity^9^, is neglected from these assay types. Therefore, it is important to be able to characterize cellular dynamics with single-cell resolution in order to delineate the many sources of cell-to-cell variation within complex biological systems.

High content screening (HCS) platforms have been used in the cancer biology field for years and their primary application has been drug discovery^10^,^11^. The novelty of high-content image-based screens over traditional high-throughput screening (HTS) platforms is the ability to acquire images, which, in addition to providing a visual representation of the experiment, is a powerful tool for further quantitative multivariate analysis. Recent biological and technical advances have led to the development of HCS assays that probe for cellular function in response to individual environmental perturbations, such as tissue extracellular matrix components^12^ and the presence of stromal cell types^2^,^13^, and make use of morphological characteristics to distinguish between cell subpopulations^14^,^15^. Although HCS platforms have typically been limited to assaying the effects of a single perturbation on a single cell-type^16^, a considerable effort has been placed on designing drug screens that implement 3D organotypic cultures, which more closely mimic the tumor microenvironment^17^,^18^. While these assays are beginning to provide significant insight into the complexities of the microenvironment, detailed methodologies capable of measuring the dynamic phenotypic responses of heterogeneous cell populations exposed to physiologically relevant tumor conditions, are largely unavailable.

Here we describe a high-throughput method that uses images from a high-content screening system to quantitatively assess heterogeneous cell populations under multiple co-occurring environmental perturbations *in vitro*. To our knowledge at time of writing, this study represents the first application of HCS for dynamic, multi-parameter analysis to quantify the heterogeneity of cellular phenotypes (i.e. cell birth, death, and changes in morphology) in 2D and 3D co-culture systems^16^. The power of this approach comes from the ability to easily and quantitatively examine cellular phenotypes across different timescales and in high-throughput providing novel insights into cellular dynamics that were overlooked when directly compared to traditional assay readouts from MTS and flow cytometry. As a result, in using this platform one can investigate a large number of conditions at multiple time points to determine “context dependent rates”, rather than sampling at a single moment in time or a single concentration value, where the outcomes might be vastly different depending on when you look. Tumors are continually adapting: what they look like today may not be what they look like tomorrow. The quantitative nature of this platform provides a more accurate representation of biological processes and can feed into additional applications (e.g., mathematical model predictions) that provide further insights into population dynamics.

## Results

The ability to quantitatively track changes in cellular phenotype over time has significant implications for understanding cancer and how to treat it. We use our novel imaging-based approach to characterize diverse cellular phenotypes across a range of microenvironmental conditions in a high-throughput, quantitative, and dynamic manner. Although the application described here is focused on lung and colorectal cancer, this platform can easily be adapted to address alternative biological questions that involve other tumor or non-tumor cell types as well as a wide range of perturbations that may influence cell behavior or population dynamics.

### High-throughput, quantitative platform for studying the behavior of cellular populations

**Figure 1:**
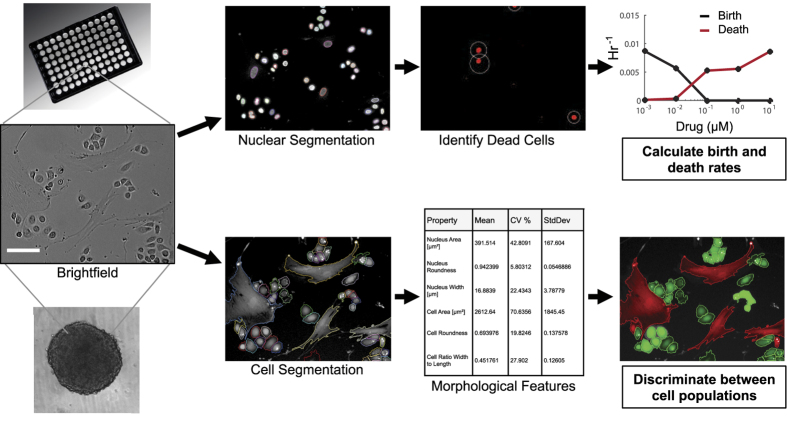
Schematic overview of experimental workflow. Images of cells in monolayer or dissociated from 3D spheroids are subjected to nuclear or cellular segmentation. Downstream analyses can include generating live and dead cell counts to calculate birth and death rates or quantification of morphology characteristics which can be used to discriminate between different cell populations. This assay design generates quantitative outputs characterizing multiple cellular phenotypes in a high-throughput manner.

Figure 1 shows a schematic of the system workflow. Cells are seeded in a multiwell plate (e.g. 96 or 384 well), and imaged in brightfield and up to four different fluorescent channels using the Operetta HCS instrument. Our automated image analysis protocol is capable of generating single cell data for millions of cells in a relatively short time frame. Depending on the type of analysis one is interested in, cells can be identified and segmented at either the nuclear or cell scale. One unique capability of nuclear segmentation is the ability to calculate live and dead cell counts over time using nuclear and dead cell stains (see methods section for experimental details). Specific filter criteria, including nuclear size and nuclei clustering, are used to count individual cells and have been optimized for each specific cell type (Supplementary Table 4). Next, the dead cell stain intensity (e.g. PI, TO-PRO-3, or DRAQ7) is calculated for all individual cells and a threshold is defined to identify those cells with stain intensities indicative of cell death (Supplementary Fig. 1). To determine the live cell count, we subtract the dead cell count from the total number of individual cells identified. The live and dead cell counts can subsequently be used to determine birth and death rates of cells as a function of microenvironmental perturbations^19^ (described in more detail in the methods section).

Another workflow that we highlight in this schematic (Fig. 1) is the ability to segment and capture additional information, including morphology features, at the cell level. In this case, specific filter criteria are used to quantify individual cells including: cell stain intensity (e.g. CellTracker Dyes), cell size, and cell roundness. We capture morphology from each individual cell and track changes in these cellular features over time. In addition, we can apply machine learning algorithms to identify and count distinct cell types (e.g. tumor cells co-cultured with fibroblasts) within a mixed population and monitor changes in cellular composition over time in an unbiased, high-throughput fashion. The advantage of our HCS approach is the ability to multiplex experimental design (i.e. assay numerous cell and environmental conditions) and data analysis (i.e. analyze multiple phenotypic parameters from a single experiment) in an automated and robust manner.

### Birth and death rates vary across cell types and microenvironments

**Figure 2:**
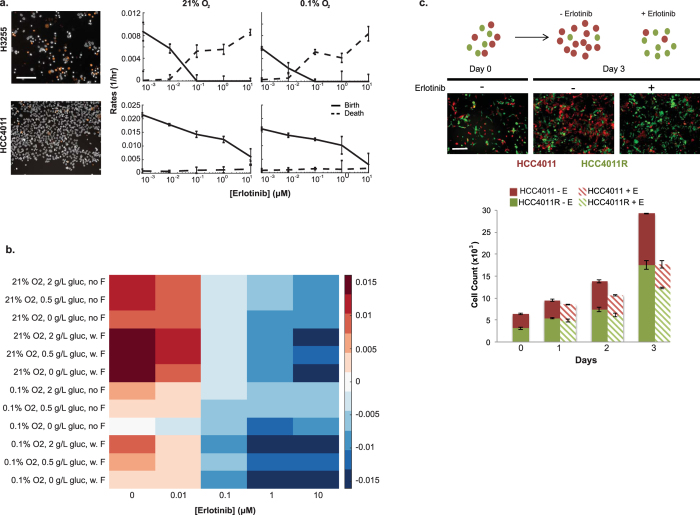
Quantitating the birth-death processes of multiple cell populations under selective pressures. **(a)** Representative images showing Hoechst (white) and propidium iodide (orange) staining of H3255 and HCC4011 cells treated with erlotinib (0.1 μM). Live and dead cell counts were obtained by segmenting nuclei and identifying dead cells based on propidium iodide intensity levels. Birth and death rates were then calculated for each cell type in response to erlotinib (0–10 μM) and oxygen perturbation (21%- normoxia and 0.1% O_2_– severe hypoxia). **(b)** Net growth rates (units = 1/hour), represented by heatmap color, of H3255 cell line exposed to 40 different microenvironmental contexts. Drug (x-axis), oxygen (O2), glucose (gluc), and fibroblasts (F) were the micreonvironmetal conditions perturbed. **(c)** HCC4011 (red) and HCC4011R (green) cells were admixed at a starting ratio of 1:1 and treated with 2 μM erlotinib (E) or control as indicated. Representative images of cell populations on day 0 and 3 in the presence or absence of drug are shown. Cell counts were generated based off of Hoechst nuclei segmentation and dead cells were identified by DRAQ7 staining. HCC4011 and HCC4011R cells were differentiated based upon their fluorescent intensity of RFP and GFP, respectively. Scale bars, 100 μm (**a,b**).

Drug treatments may elicit different cellular responses depending on concentration and cell types. This is important to consider in many applications, such as drug development, where it may be beneficial to have cells in a quiescent state as opposed to undergoing apoptosis, or vice versa^20^. Our HCS method is able to distinguish between cytotoxic and cytostatic responses to drug treatments by differentiating between a decrease in cell birth versus an increase in cell death, as depicted in Fig. 2A. While the net growth rate was slowed in both non-small cell lung cancer cell lines, H3255 and HCC4011, in response to high concentrations of the EGFR tyrosine kinase inhibitor erlotinib, our analysis identified that the HCC4011 cells displayed a steady decrease in birth rate with a minimal effect on death rate, while the H3255 cells responded in the opposite fashion with a large increase in death rate and a minimal effect on birth rate. Birth rates also decreased in both cell lines when exposed to 0.1% oxygen conditions.

Traditional viability assays, such as MTS, tend to report response to therapy data as percent viability, which in this case would have failed to capture the distinct therapeutic response between these two cell lines, a cytostatic response by HCC4011 and a cytotoxic response by H3255s (Fig. 2A, Supplementary Fig. 2). It is important to note that cell birth and death phenotypes are highly dependent on resource and space availability. Therefore, controlling for cell number in experimental assays is crucial to achieve reproducible data. There is a range of cell seeding values that will result in exponential growth during the course of an experiment; however, too few or too many cells and the growth becomes sub-exponential. Cell seeding can also affect the observed response to drug and can result in an inaccurate IC_50_ calculation (Supplementary Fig. 3). With this imaging based approach, one can be sure that an appropriate seeding density is used; a feature which is absent when performing enzymatic viability assays, yet may have a profound impact on the generated results.

Cells rarely encounter a single stimulus *in vivo.* It is the integration of multiple co-occurring factors that drives cellular phenotypes. Due to the high-throughput capacity of this platform, we are able to capture cellular responses to various microenvironmental factors individually and concurrently^19^. As shown in Fig. 2B, H3255 cells were exposed to forty different microenvironmental contexts, with perturbations including oxygen, drug, glucose, and fibroblasts. We reveal that the presence of fibroblasts increases H3255 cell growth and renders them less sensitive to low glucose concentrations in comparison to standard laboratory culture conditions (21% O2, 2 g/L glucose, no fibroblasts). In addition, we demonstrate that molecular distinctions, such as point mutations or amplifications, between cells of the same ‘type’ (e.g. erlotinib sensitive and resistant cells, H3255 and H3255R) can result in differential growth rate responses when exposed to identical selective pressures (pO2 and drug) (Fig. 2A, Supplementary Fig. 4). In particular, we highlight the importance of distinguishing between the effect of microenvironmental factors on cell birth, death, or a combination of both, which is often missing in current biological studies.

#### Using fluorescent markers to distinguish between subpopulations in co-cultures

In most physiologically relevant systems, there is often more than one cell type involved. Cell populations are in a constant flux that is governed by the evolutionary pressures being imposed on the system. Thus, it is valuable to include multiple cell types in experimental design, and, more importantly, be able to distinguish between the populations. For example, recent work has shown that clonal subpopulations of lung cancer cells resistant to erlotinib exist at a low frequency prior to treatment^21^. Therefore, it is of interest to jointly study mixed populations of cells in order to investigate the targeted effects of drug or other microenvironmental conditions that may exist on particular subpopulations. As shown in Fig. 2C, we are able to visualize and quantitatively measure the actual cell counts (live and dead) of HCC4011 sensitive and resistant subpopulations across time and under drug treatment. This analysis is carried out using a machine-learning algorithm (as described in the methods section) that allows one to train and identify live and dead, sensitive (RFP-positive) and resistant (GFP-positive) cells within a population in an unbiased fashion. Alternatively, intensity thresholds can be used to separate out populations; however, the heterogeneity in fluorescence expression across individual cells can make the multi-parameter machine-learning approach the ideal choice as it is less prone to errors (data not shown).

### Cell morphology features vary across treatments and over time

**Figure 3:**
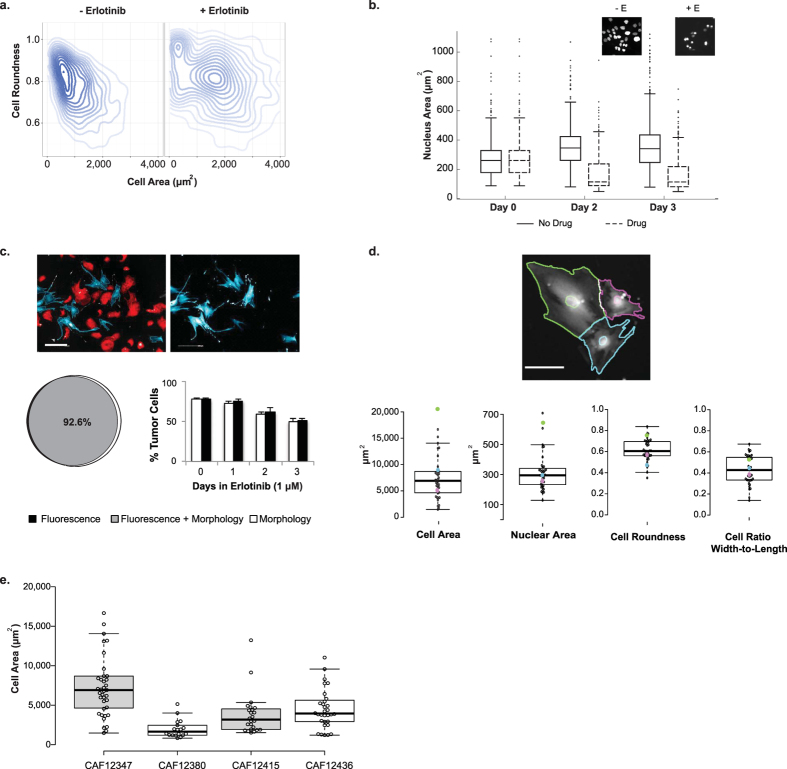
Morphological changes can be tracked over time to distinguish between populations. **(a)** Density plot displaying population distributions of cell area versus cell roundness in H3255 cells in the absence (left; n = 1,014 cells) and presence (right; n = 499 cells) of 1 μM erlotinib (E). Cells were segmented based on CellTracker orange intensity thresholds. **(b)** Nuclear area distribution was tracked over time in H3255 cells with and without 1 μM erlotinib. **(c)** *Top –* Representative images of H3255 (red) and CCD-19Lu (blue) classification as compared with labeled fibroblasts (pseudo-stained blue). *Bottom Left* – Venn diagram illustrates concordance between fluorescent (assumed truth) and morphologic classification of H3255 and CCD-19Lu, which was determined to be 92.6%. *Bottom Right* - Classification based off of morphological characteristics was evaluated against fluorescence intensity over time and in the presence of erlotinib (1 μM). **(d)** Cell morphology heterogeneity was evaluated for primary colorectal cancer associated fibroblasts isolated from an individual patient, CAF12347. Parameter values, including cell area, nuclear area, cell roundness and cell width-to-length ratio, were quantitated (n = 41) and cell segmentation mask colors correspond to parameter values for each cell displayed in boxplots. (**e)** Heterogeneity of cell area across primary colorectal cancer associated fibroblasts isolated from different patients (CAF12347, CAF12380, CAF12415, and CAF12436) was calculated. Scale bars, 200 μm (**c**) 100 μm (**d**).

Morphological cell features have proven to be important in predicting clinical outcomes and correlating with cellular signaling^8^,^9^,^15^,^22^. Using our high-content screening approach, we are able to measure and track changes in a wide range of morphological characteristics at the single cell level and quantitate these morphological feature distributions in response to microenvironmental perturbations. To highlight this application, we depict a distribution of cell areas and roundness from over two thousand H3255 cells in the presence or absence of erlotinib treatment (Fig. 3A). H3255 cells were initially identified and segmented based on the intensity of a CellTracker stain (a nontoxic, transient dye used for live-cell imaging). For each individual cell, morphology feature values were determined (Supplementary Table 5). We are able to capture the heterogeneity of morphology values within a cell population, and also show a shift in this distribution toward a larger cell area in response to drug treatment, which likely represents a subpopulation of cells that are undergoing cell death. Variation in cell morphology may also be the result of cells in different stages of the cell cycle^15^. In addition to cell synchronization by chemical or serum starvation methods, there are commercially available kits (i.e. Invitrogen Click-iT EdU HCS Assay) specific for HCS that can be used to elucidate the possible role of cell cycle stages in morphological heterogeneity.

In addition to capturing morphology changes at a singular time point, we are also able to describe fluctuations in nuclear and cell morphology over time. For example, nuclear areas for the H3255 cells tend to shrink when exposed to erlotinib. We are able to quantitate this change in nuclei area for each cell and observe how this phenotype shifts over time and across the population (Fig. 3B). We independently validated our morphology results using CellProfiler, an open source image analysis software package, and demonstrated a similar trend in nuclear and cellular area across time points and in response to erlotinib treatment (Supplementary Fig. 5). Another important aspect to this image-based approach is the multiplexing capability. For example, the morphology data described in Fig. 3B was generated from the same cell images used to determine the birth and death rate data presented in Fig. 2A. Specifically, we know from the death rate calculations in Fig. 2A, that the H3255 cells undergo significant cell death at the higher concentrations of erlotinib, which can also be observed at a morphological level with an increase in cell size (Fig. 3A) and a decrease nuclear area (Fig. 3B). Therefore, one is able to get cell morphology and cell behavior information on the same cells from a single assay, reducing variability, assay time, and reagent costs, and revealing interesting parameter associations from this multivariate analysis.

#### Using morphological features to distinguish between subpopulations in co-cultures

The importance of stromal cells, such as fibroblasts, in cancer progression has resulted in the desire to have co-culture assays where tumor and fibroblast populations can easily be distinguished. However, creating stable fluorescent cell lines, a common approach to differentiate cell types, is time consuming and may not be desirable for all cells, such as those obtained from patient samples. Here, we present an additional methodology to distinguish between populations in a co-culture assay by utilizing differences in morphological features. After pre-calculating several properties (Supplementary Table 5), the most relevant morphology features were identified following a training phase which consisted of at least 100 cells per population (larger sets achieved similar results). For the tumor (H3255) and lung fibroblast populations (CCD-19Lu), five morphology features were identified as significantly different (Supplementary Table 6) and linear coefficients were calculated for each of these properties. Automated supervised classification was then done using this information to categorize each individual cell. In a direct comparison to the fluorescence classification, the two agreed approximately 93% of the time and this concordance was consistent over time and even when drug was added (Fig. 3C, Supplementary Table 7). As a ground truth assessment, we manually annotated over 300 cells and categorized them as either tumor or fibroblast. These findings agreed with the automated analysis classifications 90% of the time (Supplementary Table 8). We used morphology features to identify and track the composition changes of the population (i.e. fluctuations in tumor and fibroblast cell percentages, which include live and dead cell counts) under various tumor selective pressures (Supplementary Fig. 6). We observed drug effects on the tumor cells, without the undesired effects on non-cancerous cells, which is an important consideration in drug development.

#### Using morphological features to assess cellular heterogeneity of primary cultures

Cellular populations derived from patient tumors are more diverse than those found in standard laboratory cell lines. The biological importance of this heterogeneity is not yet fully understood, as there is a need for further experimental methods for such investigations. Here we use the aforementioned method to investigate cancer-associated fibroblasts (CAFs) isolated from patient colon tumors. All samples were obtained after receiving informed consent from subjects and methods were carried out in accordance with approved guidelines. As visualized in Fig. 3D,E, CAFs from a single patient and across patients display strikingly different morphological features.

### Comparison of HCS to traditional cell biology assays

**Figure 4:**
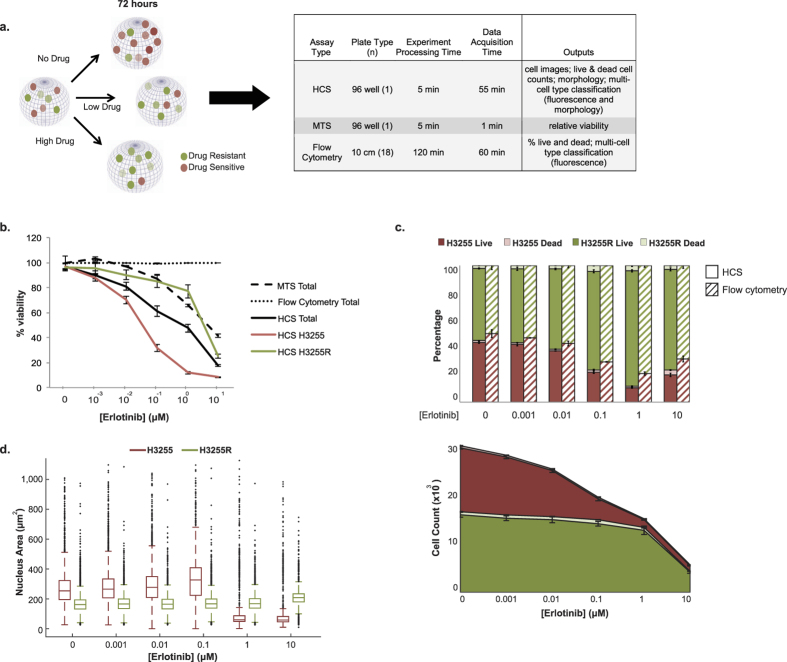
Comparison of HCS platform to standard cell biology assays, MTS and flow cytometry. **(a)** Illustrates schematic of experimental design. Cells were admixed at a starting ratio of 1:1 H3255 to H3255R-RFP cells and treated with erlotinib. Following a 72-hour incubation, cell populations were analyzed via MTS, flow cytometry, or our HCS approach. The table summarizes important features of each experimental design including processing time and data outputs. **(b)** Viability curve displaying the data generated from each assay. The flow cytometry curve remains constant across drug concentrations (at approximately 100% viability) due to loss of dead cells during sample processing and the acquisition of relative counts (not absolute counts) of live cells. **(c)** *Top* – Population percentages of H3255 live, H3255 dead, H3255R live, and H3255R dead as determined by flow cytometry and HCS analyses. *Bottom* – Absolute cell counts generated by HCS over time and in response to erlotinib treatment. **(d)** Box plot showing the distribution of H3255 and H3255R nuclear area in response to increasing concentrations of erlotinib.

We demonstrate the utility and added advantages of our HCS approach in direct comparison with common MTS and flow cytometry assays (Fig. 4). H3255 and H3255R-RFP cells were admixed at a starting ratio of 1:1 (determined via HCS to be 54% H3255 and 46% H3255R-RFP) and the three assay types were set up side-by-side (Fig. 4A). As shown in Fig. 4B, MTS is unable to differentiate between the two cell populations (i.e. sensitive versus resistant) nor discriminate between cells that are alive and growth arrested versus dead, resulting in a erlotinib viability curve that more closely resembles the resistant population. The discordant percent viability of the total population between MTS, flow cytometry, and HCS may be due to: assay sensitivity (i.e. MTS - colorimetric absorbance readout; flow cytometry - defined event number; HCS - absolute cell count), loss of dead cells during sample preparation (flow cytometry), or distinctive metabolic activities between cell types (MTS). Analysis of tumor population composition was comparable between HCS and flow cytometry (Fig. 4C, top); however, flow cytometry detected less than 0.01% dead cells, likely due to their loss while preparing the samples. Although flow cytometry reveals an increasing overall percentage of the H3255R population as erlotinib concentration increases, it is through HCS that we determine that the resistant population counts are in fact not increasing, but the overall population size is actually decreasing (Fig. 4C, bottom). In a clinical setting, it is important to not only detect changes in the total population size, but also the cellular composition. A decrease in tumor size could be mistaken for successful treatment, yet if the remaining population is completely resistant to the current therapy, the prognosis may be poor depending on alternative treatments available.

In addition to generating live and dead cell counts of the H3255 and H3255R populations as a function of erlotinib concentration (Fig. 4C, bottom), with the same HCS experimental setup we calculate morphology features on a single-cell level (Fig. 4D). There is a distinct difference in nuclear area between the sensitive and resistant subpopulations in addition to changes in size over time as a result of drug treatment, with H3255 cells showing a more dramatic shrinkage in nuclear size as drug concentration is increased. The additional features of HCS provide a more accurate, detailed representation of each subpopulation’s response to increasing drug. The advantages of our HCS approach, including time saved and outputs generated, are summarized in Fig. 4A. The utility of MTS and flow cytometry assays is limited, especially in heterogeneous systems such as cancer.

### Quantitating changes in cellular composition in 3D cultures

**Figure 5:**
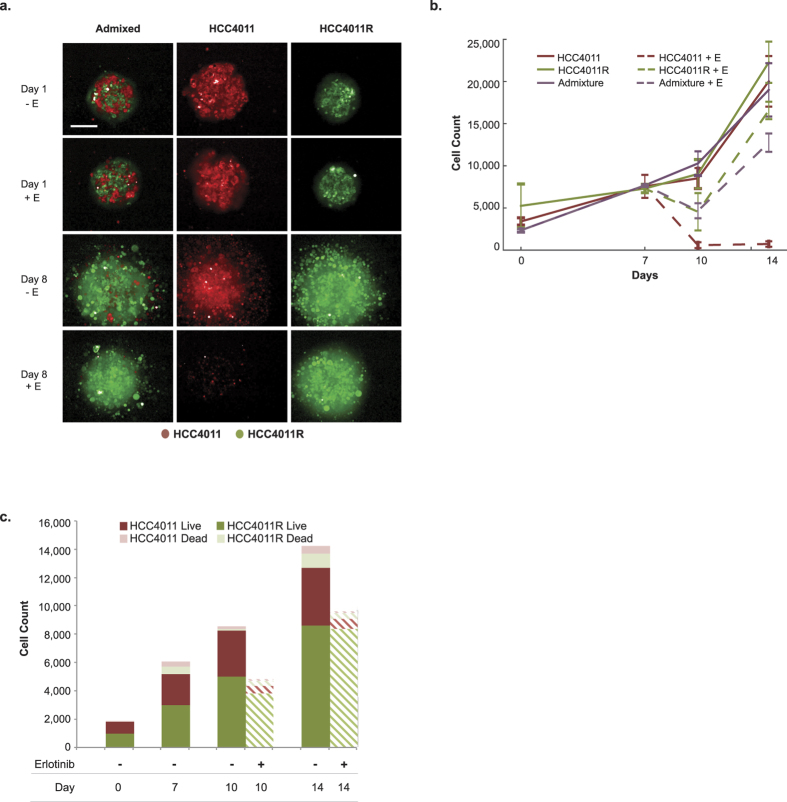
Evaluation of tumor composition in 3D. (**a**) Representative maximum projection images of HCC4011 (red), HCC4011R (green), and admixed (1:1 ratio, HCC4011:HCC4011R) spheroids on days 1 and 8 with and without erlotinib (1 μM) treatment. Spheroids were dissociated on days 0,7,10, and 14 and monolayer cultures were stained with Hoechst and DRAQ7 and imaged to generated live and dead cell counts. (**b**) Average cell count from dissociated tumors was plotted over time for HCC4011, HCC4011R, and admixed tumors. Spheroids were treated with erlotinib on day 7. (**c**) Bar graph depicting average quantitative changes in HCC4011 and HCC4011R subpopulations (live and dead cell counts) in admixed spheroids over time in response to erlotinib treatment (1 μM).

3D cultures provide a more realistic *in vitro* model for studying tumor cell populations, with gradients of oxygen and nutrients naturally occurring. Therefore, it is of interest to extend our quantitative analysis from 2D cultures to a more physiologically relevant model system. We developed a high-throughput spheroid dissociation protocol (see methods section) to enable quantitation of individual cells and their respective phenotype (e.g. live vs. dead). While this digestion process may introduce possible bias, it is advantageous over current 3D quantitative applications because of its novelty in providing high-throughput capabilities without requiring specialized microscopy instrumentation. Figure 5A depicts representative spheroid images of unmixed and admixed subpopulations of sensitive and resistant cells over time and in the presence and absence of erlotinib treatment. In Fig. 5B, we display total cell counts from spheroids of erlotinib sensitive, resistant, and admixed cell populations at multiple time points and in response to therapy (1 μM erlotinib). Cell counts for each subpopulation were obtained by segmenting nuclei and using a linear classifier with four classes (HCC4011 live, HCC4011 dead, HCC4011R live, HCC4011 dead) based on fluorescence intensities of RFP (HCC4011), GFP (HCC4011R), and DRAQ7 (+dead; - live) as well as morphology features. The linear classifier was trained on pure subpopulations to determine the rate of correct classification and then applied to the admix samples. The machine learning was considered acceptable once the control cells were correctly classified at least 90% of the time.

While qualitative information regarding tumor composition is obtained by visualizing the relative ratio of GFP to RFP cells (Fig. 5A), a unique aspect of our platform is the ability to generate quantitative data for each population in a high-throughput, robust, and unbiased manner (Fig. 5C). Here we monitor the relative fitness of individual subpopulations within a heterogeneous 3D culture comprised of oxygen, nutrient, and drug gradients. We use the 4-class linear classifier to determine the cell counts from each subpopulation within an admix spheroid and to track changes in these cell counts over time during spheroid growth or as a result of treatment (Fig. 5C). We are able to observe HCC4011R cells outgrowing HCC4011 cells even in the absence of drug.

## Discussion

Our quantitative, image-based HCS platform improves upon current cell viability assays by providing a more comprehensive picture of diverse cellular dynamics within the contexts of a heterogeneous tumor microenvironment (Fig. 4). Due to the high-throughput nature of this approach, we are able to explore a variety of physiologically relevant selective pressures (including different types and quantities), resulting in tens to hundreds of conditions probed simultaneously. Here we highlight the unique ability to measure the temporal dynamics of multiple phenotypic properties, specifically growth rate and morphology features, that are known to be important in cancer progression^23^,^24^. With our platform, we can track cellular fitness dynamics being driven by evolutionary selective pressures that more closely mimic a tumor, which has a direct impact on the tumor composition. Knowledge of such dynamical changes in cellular heterogeneity and treatment response (e.g. changes in drug resistant fraction of cells) may have a significant clinical impact at the time of treatment by influencing drug choice and dosing strategy.

Our system has several advantages for scientists in the cancer biology field. On the biological side, it allows one to (1) investigate phenotypic contributions at the single cell or subpopulation level, capturing the heterogeneity of tumors; (2) perform more complex experiments that encapsulate the relevant cellular and environmental features of a tumor; and (3) conduct a more thorough multidimensional analysis of the data. On the technical side, our platform, in comparison to traditional viability assays, has the ability to visualize cells to identify whether confounding issues (i.e. seeding error, contamination, morphology changes) may have occurred. Further, the enzymatic reactions that assays such as MTS rely on may not be accurate in some microenvironmental contexts, such as varying glucose conditions^7^, and are unable to differentiate between multiple cell populations, which limits their ability to probe heterogeneous systems. Specifically, we show drastic differences in cell viability outcome when directly evaluating results of an erlotinib-treated co-culture of drug sensitive and resistant cells acquired by HCS compared with MTS and flow cytometry assays. These differences can be attributed to the shortcomings of traditional assays and include: the inability to distinguish between two cell populations, an incomplete phenotypic characterization (cell birth versus death), and technical challenges associated with assay preparation (Fig. 4).

The potential of HCS platforms extend beyond what is discussed here. Previous work has demonstrated the utility of quantitative imaging to investigate cell division rates^5^, calculate drug treatment response^25^, track individual cell kinetics^26^, and elucidate molecular signaling dynamics^27^. For applications such as live-cell imaging of 3D organotypic cultures or 2D intracellular dynamics, one may want to compromise speed and high-throughput capacity to achieve better image resolution or depth, and therefore may consider other imaging platforms (e.g., two photon confocal microscopy) with capabilities that better suite these specific needs. Finding a balance between the speed and volume of data from a HCS platform compared to the resolution and depth provided by other imaging platforms is something researchers will have to keep in mind during experimental planning. It is also essential to acquire images of good quality to achieve the most from further downstream analyses. If cells grow in clumps rather than in a uniform layer, nuclear segmentation and subsequent morphological analyses may not be possible. If cell populations are not morphologically distinct or are densely compact, automated classification may be bias. Other important aspects to consider during experimental design and analysis are reviewed in Boutros *et al*.^11^. We also recognize that HCS platforms are not available in all research labs or institutions, and therefore stress that the described protocols can be accomplished on a smaller scale using any imaging platform coupled with analysis protocols generated from open source software (i.e. CellProfiler^28^, ImageJ) or other high content analysis software. Here we illustrate comparable morphological outputs (nuclear and cell area) obtained from CellProfiler and Harmony.

A considerable push in the cancer community is the use of patient samples and 3D culture models (e.g. circulating tumor cells, patient-derived xenografts, and organoids) in preclinical studies^17^,^18^,^29^. Along with the benefits of using patient derived material come the technical hurdles centered on the complexity of its composition. Therefore, the ability to distinguish between cellular populations based on fluorescence or morphological features as described here (Fig. 3D,E) may help mitigate some of the technical challenges with patient samples. Also, given the high-throughput nature of this approach, one can perform large screens on cells directly from patient biopsies (tumor and host) to identify which drugs are most effective in causing cell death, a reduction in cell birth, or changes in morphological properties indicative of response, thereby providing a personalized medicine approach. The most promising treatment strategies can then be validated *in vivo* before being introduced into the clinic.

The quantitative and standardized nature of HCS datasets allows for amenable integration into physical science applications such as mathematical modeling of population dynamics (Supplementary Fig. 7) or emerging data formats (e.g. generation of digital cell lines – MultiCellDS, www.multicellds.org). Integration of the physical and biological sciences has proven to be an effective interdisciplinary approach for understanding cancer progression and identifying novel treatment strategies^30^,^31^,^32^,^33^, and we believe this quantitative imaging platform coupled with computational modeling will help further advance this field. This platform is a powerful tool to rapidly screen multiple drug and microenvironmental conditions simultaneously and determine their effects on a heterogeneous population of patient-derived cells in order to design personalized treatment strategies.

## Methods

### Cell culture and reagents

CCD-19Lu (lung fibroblasts) were acquired from ATCC and maintained in EMEM media. NSCLC isogenic erlotinib sensitive and resistant (R) cell lines (HCC4011, HCC4011R-Met amplified; H3255, H3255R-T790M; PC9, and PC9R-T790M; HCC827, and HCC827R-T790M) were acquired from Dr. William Pao (while at Vanderbilt University) and cultured in RPMI 1640 media (see Supplementary Table 1). All culture media was supplemented with 10% fetal bovine serum (Gemini) and 1% penicillin/streptomycin solution and cells were kept under standard laboratory conditions (5% CO_2_, 37 °C) unless otherwise noted. Erlotinib resistant cell lines were derived through dose escalation methods, as described in Ohashi *et al*.^34^, and were maintained in 1 μM erlotinib (LC Laboratories # E-4007). Fluorescent cell lines were generated by transduction with MISSION pLKO.1-puro-CMV-Turbo GFP or RFP lentivirus particles (Sigma SHC003V, SHC012V) and maintained in 1 μg/mL puromycin (Life Technologies #A11138-03). For primary cell isolation, colon tumors were washed in PBS and minced using two scalpels. Tumors were then digested with 1.5 mg/mL collagenase, 20 μg/mL hyaluronidase, and 10 μM Ly27632. Cells were incubated for 30 minutes, strained, and washed with PBS. Primary cell cultures were grown in DMEM/F12 supplemented with 10% FBS and 1% penicillin/streptomycin. Experimental protocols utilizing patient samples were approved by the University of Southern California Health Science Campus Institutional Review Board (IRB protocol number HS-06-00678).

### Experimental culture conditions

For HCS experiments, cells were seeded at either 4,000 (HCC4011, H3255) cells per well at least eighteen hours prior to drug treatment on 96-well plates (Corning #3904). For a standard assay setup, one plate per time point was seeded (all at the same time) and the addition of dyes resulted in endpoint analysis. For hypoxia assays, cells were directly placed in a Biospherix C-Chamber set at 0.1% O_2_ following seeding and subsequent work was done in a hypoxia glove box to maintain constant oxygen control. For glucose modulation, glucose-free media (0 g/L) was supplemented with D-(+)-Glucose solution (Sigma, G8644) to achieve the desired concentration. Prior to imaging, cells were stained with 5 μg/mL Hoechst 33342 (Invitrogen #H21492) and either 5 μg/mL Propidium Iodide (PI) (Invitrogen #P1304MP), 5 μg/mL TO-PRO-3 Iodide (Life Technologies T3605), or 5 μM DRAQ7 (Biolegend, #424001) to identify cells as live or dead, respectively, depending on the fluorescent channels being used for imaging. For morphology assays, cells were stained with 10 μM CellTracker dye (Life Technologies, Orange CMRA #C34551) for thirty minutes and washed with PBS to reduce background.

### Tumor spheroid generation and digestion

Monolayer HCC4011 and HCC4011R cells were trypsinized, re-suspended in RPMI 1640 growth media and filtered using a 70μm filter. Cells were counted three times and counts were averaged. 2000 cells per well were seeded in either a 96-well Perfecta3D^®^ hanging drop plate (3D Biomatrix, #HPD1096) for spheroid formation, or a 96 well flat bottom plate for an initial seeding count. Flat bottom plates were incubated overnight and cells were stained with Hoechst 33342 and DRAQ7 (dead cell stain) for 30 minutes prior to imaging. Hanging drop plates were incubated for one week at 37 °C, 5% CO_2_ to allow for spheroid formation. On Day 0 (i.e. 7 days post cell seeding) hanging drop spheroids were transferred to a CellCarrier™-96 Spheroid ULA/CS plate (Perkin Elmer, 6055330). Fresh media or 1 μM erlotinib was added to each well and the plate was incubated at 37 °C.

On days 0, 3, and 7, a respective number of spheroids were transferred to a separate 96 well U-bottom plate, and centrifuged at 3000 RPM for 5 minutes at 4 °C. Growth media was gently removed and spheroids were washed in PBS 2x, centrifuging between each wash. PBS was gently removed and 50 μl digestion media was added to each well (cocktail of: 1% Collagenase Type II 50mg/ml (Millipore, #234155), 72% Trypsin:EDTA (0.25%), and 27% PBS) and plates were incubated at 37 °C for 10 minutes. 50 μl fresh digestion media was added and each well was mixed using a multichannel pipette. Spheroids were incubated for additional 10 minutes. 100 μl growth media was added to each well and mixing was performed again. The plate was centrifuged at 3,000 RPM for 5 minutes at 4 °C. Supernatant was aspirated and cells were re-suspended in 200 μl growth media. A 96-well flat bottom plate was filled with 175 μl growth media per well. 25 μl of cell suspension was placed in each well. The plate was incubated overnight to allow the cells to adhere and prepared for imaging in the same manner as the seeding count plate described previously.

### Image acquisition

#### Monolayer Experiments

Images were acquired on an Operetta High Content Screening (HCS) System (Perkin Elmer) equipped with climate control (37 °C, 5% CO_2_) using a 10X objective lens. Each condition was assayed in at least triplicate wells and a minimum of 24 fields per well per time point (0, 48, 72 hours post drug addition) were imaged. Additional details regarding the imaging protocols used for each cell line can be found in Supplementary Tables 2 and 3.

#### Tumor Spheroid Experiments

Intact spheroids were imaged on the Operetta HCS in confocal mode using the z-stack function. Ten planes were imaged at intervals of 3 μm for a total height of 30 μm.

#### Image analysis. 

Image analysis was performed using the Harmony 3.5.2 software (Perkin Elmer) (or CellProfiler 2.1.1 when stated^28^). All data points considered for analysis were taken before any confluence effects were apparent.

#### Calculation of Birth and Death Rates

Birth and death rates were calculated using time series data consisting of live and dead cell counts obtained over a period of 0–72 hours under various environmental perturbations as previously described^19^,^32^. To determine live and dead cell counts, nuclei were segmented using the Hoechst channel or GFP channel (for cells labeled with histone-2B-GFP, Supplementary Fig. 8). Propidium Iodide, TO-PRO-3 Iodide, or DRAQ7 intensity was used to classify cells as dead. For Propidium Iodide, cells were identified as dead based upon an intensity threshold (Supplementary Fig. 1). For the dead cell stains in the far red channel (TO-PRO-3 Iodide and DRAQ-7), the background intensity −30 pixels (+/−15, depending on cell type) outside the nucleus was measured, and cells with a nuclear to background ratio greater than 1.2 were classified as dead (Supplementary Fig. 1). To obtain net growth rates (birth minus death rates), the experimental counts of live cells at various time points under each microenvironmental perturbation were fit to an exponential growth model. A linear regression of the log-transformed data was performed to obtain fitted rates at each drug concentration. Using the assumption of a constant death rate per live-cell unit of time, death rates were calculated using the accumulated amount of cell deaths over the time period. The cell birth rate was then equated to the sum of the net growth rate and the death rate for each cell population under the microenvironmental conditions perturbed.

#### Calculation of Cell Morphology Features

Nuclei were segmented using the Hoechst channel and cytoplasmic boundaries were segmented based off of CellTracker stain (Supplementary Tables 4–6). Cell morphology features were calculated on a single-cell level. Cells with poor segmentation were excluded.

#### Cellular Classifications Based on Fluorescence or Morphology Features

In order to classify fluorescent cells into subpopulations (e.g. GFP-labeled erlotinib resistant versus RFP-labeled erlotinib sensitive cells), intensity thresholds were applied to filter out cells that did not express the fluorescent label of interest. To classify cells based on differences in morphology, cells were segmented as described above. Data sets were then subjected to linear classification analysis (PhenoLOGIC). Briefly, training sets consisting of 100 cells per population were generated and thirty parameter values for various morphological properties were calculated for every cell to identify the most relevant properties for cell classification (Supplementary Table 5). For manual classification to determine ground truth, >300 cells were blindly classified into subpopulations based on appearance and these results were compared to the automated classification (Supplementary Table 8).

#### MTS assay

H3255 sensitive and resistant cells were admixed at a ratio of 1:1 and seeded at 4,000 cells per well (in Costar 96-well plates, #3596) in phenol-red free RPMI (Gibco, #11835-030). Twenty-four hours after seeding, cells were treated with erlotinib at the designated concentrations and incubated for 72 hours. CellTiter 96 AQ_UEOUS_ One Solution Cell Proliferation MTS Assay (Promega G3582) was then performed following manufacturer’s instructions and plates were read on SpectraMax M5 Microplate Reader (Molecular Devices). All data points were performed in triplicate.

#### Flow cytometry

H3255 sensitive and resistant cells were admixed at a ratio of 1:1 and seeded at 2.2 × 10^6^ cells per well in 10 cm plates. Twenty-four hours after seeding, cells were treated with desired concentrations of erlotinib and incubated for 72 hours. Cells were trypsinized, washed with PBS, and stained with Hoechst and DRAQ7. Samples were run in triplicate on a BD FACSAria.

## Additional Information

**How to cite this article**: Garvey, C. M. *et al*. A high-content image-based method for quantitatively studying context-dependent cell population dynamics. *Sci. Rep.* **6**, 29752; doi: 10.1038/srep29752 (2016).

## Supplementary Material

Supplementary Information
