# Supplementary material for: A high-content image-based method for quantitatively studying context-dependent cell population dynamics
Source: Sci Rep. 2016 Jul 25;6:29752. doi: 10.1038/srep29752 (PMC4958988; doi:10.1038/srep29752)

SUPPLEMENTARY INFORMATION

Title of Manuscript:

A high content image-based method for quantitatively studying context-dependent cell population dynamics

Authors:

Colleen M. Garvey[1], Erin Spiller[1], Danika Lindsay[2], Chun-Te Chiang[1], Nathan C. Choi[1], David B. Agus[1], Parag Mallick[3], Jasmine Foo[2], Shannon M. Mumenthaler[1]†

[1]Lawrence J. Ellison Institute for Transformative Medicine, University of Southern California, Los Angeles, California.
[2]School of Mathematics, University of Minnesota, Minneapolis, Minnesota
[3]Department of Radiology, Canary Center at Stanford for Cancer Early Detection, Stanford University, Palo Alto, California

† To whom correspondence should be addressed

Supplementary Methods

Experimental conditions:
For live cell imaging, PC9 cells were transduced with Histone H2B-GFP Lentiviral Biosensor (EMD Millipore 17-10229) and FACS sorted for GFP positive cells. PC9 cells were seeded at 1,500 cells/well and HCS experiments were performed as described in the main methods section of the manuscript.

Mathematical model description:
We employ a continuous time multi-type branching process to model the tumor cell population, which is comprised of drug-sensitive and drug-resistant cell populations. In this model, each individual cell waits a random exponential amount of time to divide or die; the exponential rate parameters are specified by cellular birth and death rates. These birth and death rates are dynamic and may vary with cell type and current drug concentration. Each sensitive cell division may give rise to a resistant daughter cell with a probability of 10^{-7} mutations per cell division[32]. For any dosing regimen, the probability of developing resistance by a specific time and expected size of the resistant population can be determined (see our previous work[33]). All model parameters (birth and death rates) are measured experimentally in a spectrum of drug concentrations as described above.

Supplementary Results

Cell counts over time use live-cell imaging:
With histone-2B-GFP labeling we are able to follow the same cells over time, which reduces the amount of reagents and setup time required, and also decreases seeding biases. As shown in **Supplementary Figure 8**, PC9 cell counts were obtained from the same 96-well plate over a seven-day time span.

Mathematical model predictions of tumor growth dynamics:
The quantitative data generated by this imaging platform can directly parameterize a mathematical model, which allows for a deeper understanding of the evolutionary processes occurring as a result of tumor microenvironmental perturbations. To exemplify this, the birth and death rates of the H3255 and H3255R cell lines were fed into a two-type birth and death process model, described in [29], to predict tumor population dynamics in response to different concentrations of erlotinib (**Supplementary Figure 7**). We illustrate differential response dynamics of the sensitive and resistant tumor cell populations to erlotinib concentrations of 0.01 µM and 0.1 µM over time. We observe that the overall increase in total tumor population size is similar between the two cases. However, the model predicts distinct evolutionary dynamics of the subpopulations comprising this 'tumor' when dosed at 0.01 vs 0.1 µM erlotinib. In particular, at the lower drug concentration the drug-sensitive population comprises a large portion of the tumor on this time scale, whereas the drug-sensitive population is negligible at higher concentrations. This exemplifies how refined quantitative data from this platform can power mathematical models to yield unique insights about tumor evolutionary dynamics that would not otherwise be accessible.

Supplementary Tables

Supplementary Table 1: Cell Type, TME Parameter Values, Drug Response. Table displaying cell types used and their IC_{50} to erlotinib and the tumor microenvironmental parameter values that were assayed.

Cell Name	Type	Erlotinib IC_{50}
HCC4011	NSCLC	0.03 μM[31]
HCC4011R (Met+)	NSCLC	>5 μM[31]
H3255	NSCLC	0.04-0.09 μM [34, 35]
H3255R (T790M)	NSCLC	>5 μM[34, 35]
HCC827	NSCLC	0.005 μM[31]
HCC827R (T790M)	NSCLC	>5 μM[31]
PC9	NSCLC	0.03- 0.15 μM[35, 36]
PC9R (T790M)	NSCLC	>5 μM[35, 36]
CCD-19Lu	Lung Fibroblast	
CAF12347	Colorectal cancer CAF	
CAF12380	Colorectal cancer CAF	
CAF12415	Colorectal cancer CAF	
CAF12436	Colorectal cancer CAF	
**TME Perturbations**	**Parameter Values**	
Erlotinib	$10^{-3} – 10^{1}$ μM	
Oxygen	21%, 0.1%	

Supplementary Table 2: Image Acquisition Parameters. Fluorescent channel filters used for image acquisition on the Operetta HCS imaging system.

Channels	Excitation Wavelength	Emission Wavelength
Hoechst 33342	360-400	410-480
GFP	460-490	500-530
Brightfield	Transmission	650-760
Propidium Iodide	520-550	580-650
RFP	520-550	560-630
DRAQ7	620-640	650-760

Supplementary Table 3: Image Acquisition Protocols. Imaging protocols used to acquire images was optimized for each cell type and excitation times for each channel and imaging height are displayed.

Cells	Height	BF	Hoechst	PI	Alexa 546	eGFP	Alexa 647 LB
HCC4011 + HCC4011R	10 um	20 ms	100 ms	200 ms	200 ms	100 ms	160 ms
H3255 + H3255R	10 um	20 ms	100 ms	200 ms			

Supplementary Table 4: Nuclei Segmentation Protocols. Designated protocols to identify nuclei following image acquisition for each cell type.

Harmony						
Cells	Method	Splitting Coefficient	Intensity Threshold	Diameter	Nuclei Area	Nuclei Roundness
HCC4011 +HCC4011R	M	0.45	0.1	20 um	>25, <900	>0.6
H3255 +H3255R	M	0.15	0.2	20 mm	>25, <1500	> 0.7
**CellProfiler**						
**Min,Max diameter**	**Threshold strategy**	**Thresholding method**	**Two or three classes**	**Threshold correction factor**	**Distinguish clumped objects**	
20,80	Global	Otsu	Three classes	1	Shape	

Supplementary Table 5: Cell Morphology Properties Calculated. Morphological properties calculate on a single cell level that were then used to differentiate between cell types.

Nucleus Area
Nucleus roundness
Nucleus width
Nucleus length
Cell area
Cell roundness
Cell width
Cell length
Cell ratio width to length
Cell symmetry 02
Cell symmetry 03
Cell symmetry 04
Cell symmetry 12
Cell symmetry 13
Cell symmetry 14
Cell symmetry 15
Cell Threshold Compactness 30%
Cell Threshold Compactness 40%
Cell Threshold Compactness 50%
Cell Threshold Compactness 60%
Cell Axial Small Length
Cell Axial Length Ratio
Cell Radial Mean
Cell Radial Relative Deviation
Cell Profile 1/5
Cell Profile 2/5
Cell Profile 3/5
Cell Profile 4/5
Cell Profile 5/5

Supplementary Table 6: Cell Morphology Properties Differentiating H3255 and CCD-19Lu. Cell morphology features that were used to differentiate between the two cell types, H3255 and CCD-19Lu cells.

Properties (ordered by relevance)	Linear Coefficient
Cell Profile 1/5	-10.6396
Cell Roundness	-3.3758
Cell Width [µm]	0.0214552
Cell Radial Relative Deviation	3.3537
Cell Symmetry 05	4.497914

Supplementary Table 7. Pure Population Identification Accuracy. Pure populations of H3255 and CCD-19Lu were subjected to analysis to differentiate the two cell lines, and the accuracy of the protocol was established.

Cell Type	# of cells	H3255	CCD-19Lu
CCD-19Lu	413	7.43%	92.57%
H3255	5,587	96.70%	3.33%

Supplementary Table 8. Validation of Linear Classifier on Admixture Samples. Following a training phase on pure H3255 and CCD-19Lu cell types, single cells in admixture populations were classified by eye and then compared to the results of the linear classifier. The two were in agreement over 90% of the time.

Object No	Manual Assessment		Linear Classifier		Agree?
	H3255	CCD-19Lu	H3255	CCD-19Lu	
1	1		1		Y
2		1		1	Y
3		1		1	Y
4	1		1		Y
5	1		1		Y
6	1		1		Y
7	1		1		Y
8	1		1		Y
9	1		1		Y
10	1		1		Y
11	1		1		Y
12	1		1		Y
13	1		1		Y
14		1		1	Y
15	1		1		Y
16	1		1		Y
17	1		1		Y
18	1		1		Y

19		1		1	Y
20	1		1		Y
21	1		1		Y
22	1		1		Y
23	1			1	N
24	1		1		Y
25	1		1		Y
26		1		1	Y
27	1		1		Y
28	1		1		Y
29	1		1		Y
30	1			1	N
31	1		1		Y
32	1		1		Y
33		1	1		N
34	1		1		Y
35	1		1		Y
36	1			1	N
37	1		1		Y
38		1		1	Y
39	1		1		Y
40	1		1		Y
41		1		1	Y
42	1			1	N
43		1		1	Y
44		1		1	Y
45	1		1		Y
46		1	1		N
47	1		1		Y
48		1		1	Y
49	1		1		Y
50	1		1		Y
51	1		1		Y
52	1		1		Y
53	1		1		Y
54	1		1		Y
55		1		1	Y
56	1		1		Y
57	1		1		Y
58	1		1		Y
59	1		1		Y
60		1		1	Y
61	1		1		Y
62	1			1	N
63	1		1		Y
64	1		1		Y
65	1		1		Y
66	1		1		Y
67	1		1		Y
68		1		1	Y
69		1		1	Y
70	1		1		Y
71		1		1	Y
72	1		1		Y
73	1		1		Y
74		1		1	Y

75	1		1		Y
76	1		1		Y
77	1		1		Y
78		1		1	Y
79	1		1		Y
80		1		1	Y
81		1		1	Y
82	1		1		Y
83	1		1		Y
84	1		1		Y
85	1		1		Y
86	1		1		Y
87	1		1		Y
88	1		1		Y
89	1		1		Y
90	1		1		Y
91	1		1		Y
92	1		1		Y
93	1		1		Y
94	1		1		Y
95	1		1		Y
96		1		1	Y
97	1		1		Y
98	1		1		Y
99	1		1		Y
100	1		1		Y
101		1	1		N
102	1			1	N
103	1		1		Y
104	1		1		Y
105	1		1		Y
106	1		1		Y
107	1		1		Y
108	1			1	N
109		1		1	Y
110	1		1		Y
111	1			1	N
112	1		1		Y
113		1		1	Y
114		1		1	Y
115	1		1		Y
116	1		1		Y
117		1		1	Y
118	1		1		Y
119		1		1	Y
120		1	1		N
121	1			1	N
122		1		1	Y
123	1		1		Y
124		1		1	Y
125	1		1		Y
126	1		1		Y
127	1		1		Y
128	1		1		Y
129		1		1	Y
130	1		1		Y

131	1		1				Y
132		1			1		Y
133		1			1		Y
134		1			1		Y
135	1				1		N
136	1		1				Y
137	1		1				Y
138	1		1				Y
139	1		1				Y
140	1		1				Y
141	1		1				Y
142	1		1				Y
143	1		1				Y
144	1		1				Y
145	1				1		N
146	1		1				Y
147	1		1				Y
148	1		1				Y
149	1		1				Y
150	1		1				Y
151		1			1		Y
152	1		1				Y
153	1		1				Y
154		1			1		Y
155	1		1				Y
156		1			1		Y
157	1				1		N
158		1				1	Y
159	1			1			Y
160	1			1			Y
161	1			1			Y
162	1			1			Y
163	1			1			Y
164	1			1			Y
165	1			1			Y
166	1			1			Y
167		1				1	Y
168	1			1			Y
169		1		1			N
170		1				1	Y
171	1			1			Y
172	1			1			Y
173	1			1			Y
174		1				1	Y
175	1			1			Y
176	1			1			Y
177	1			1			Y
178	1			1			Y
179	1					1	N
180	1			1			Y
181		1				1	Y
182		1		1			N
183	1			1			Y
184		1				1	Y
185	1			1			Y
186	1			1			Y

187	1		1		Y
188	1		1		Y
189	1		1		Y
190	1		1		Y
191	1		1		Y
192	1			1	N
193	1		1		Y
194	1		1		Y
195	1			1	N
196	1		1		Y
197	1		1		Y
198	1		1		Y
199	1		1		Y
200	1		1		Y
201	1		1		Y
202	1		1		Y
203	1		1		Y
204	1		1		Y
205	1			1	N
206	1		1		Y
207	1		1		Y
208	1		1		Y
209	1		1		Y
210		1		1	Y
211	1		1		Y
212		1		1	Y
213		1		1	Y
214		1		1	Y
215		1	1		N
216	1		1		Y
217		1		1	Y
218	1		1		Y
219	1		1		Y
220	1		1		Y
221	1		1		Y
222	1		1		Y
223		1		1	Y
224	1		1		Y
225	1		1		Y
226		1	1		N
227		1		1	Y
228		1		1	Y
229	1		1		Y
230		1		1	Y
231	1		1		Y
232	1		1		Y
233	1		1		Y
234	1			1	N
235	1		1		Y
236		1		1	Y
237	1		1		Y
238	1		1		Y
239	1		1		Y
240	1		1		Y
241	1		1		Y
242	1		1		Y

243	1		1		Y
244		1	1		N
245		1		1	Y
246		1		1	Y
247		1		1	Y
248		1		1	Y
249	1		1		Y
250	1		1		Y
251	1		1		Y
252	1		1		Y
253	1		1		Y
254	1			1	N
255	1		1		Y
256	1		1		Y
257		1	1		N
258	1		1		Y
259	1		1		Y
260	1		1		Y
261	1		1		Y
262		1		1	Y
263	1		1		Y
264	1		1		Y
265	1		1		Y
266	1		1		Y
267	1		1		Y
268	1		1		Y
269	1		1		Y
270		1		1	Y
271	1		1		Y
272	1		1		Y
273	1		1		Y
274	1		1		Y
275		1	1		N
276	1		1		Y
277		1		1	Y
278	1		1		Y
279		1		1	Y
280		1		1	Y
281	1		1		Y
282		1		1	Y
283	1		1		Y
284		1		1	Y
285		1		1	Y
286	1		1		Y
287		1		1	Y
288	1		1		Y
289	1		1		Y
290	1		1		Y
291	1		1		Y
292		1		1	Y
293		1		1	Y
294	1		1		Y
295		1		1	Y
296		1		1	Y
297		1		1	Y
298	1		1		Y

299	1		1		Y
300		1	1		N
301	1		1		Y
302	1		1		Y
303	1		1		Y
304	1		1		Y
305	1		1		Y
306	1		1		Y
307		1		1	Y
308		1		1	Y
309	1		1		Y
310		1		1	Y
311	1		1		Y
312	1		1		Y
313		1	1		N
314	1		1		Y
315		1		1	Y
316		1		1	Y
317	1		1		Y
318		1		1	Y
319	1			1	N
320		1		1	Y
321	1		1		Y
322	1		1		Y
323		1	1		N
324	1		1		Y
325	1		1		Y
326	1		1		Y
327	1		1		Y
328	1		1		Y
329	1		1		Y
330	1		1		Y
331		1		1	Y
332	1		1		Y
333	1		1		Y
334	1		1		Y
335	1		1		Y

Supplementary Figure 1 - Intensity thresholds define dead populations. Each point represents a single cell with the calculated intensity for (a) propidium iodide or (b) TOPRO-3. Dead cells were identified based upon residing above the intensity threshold set.

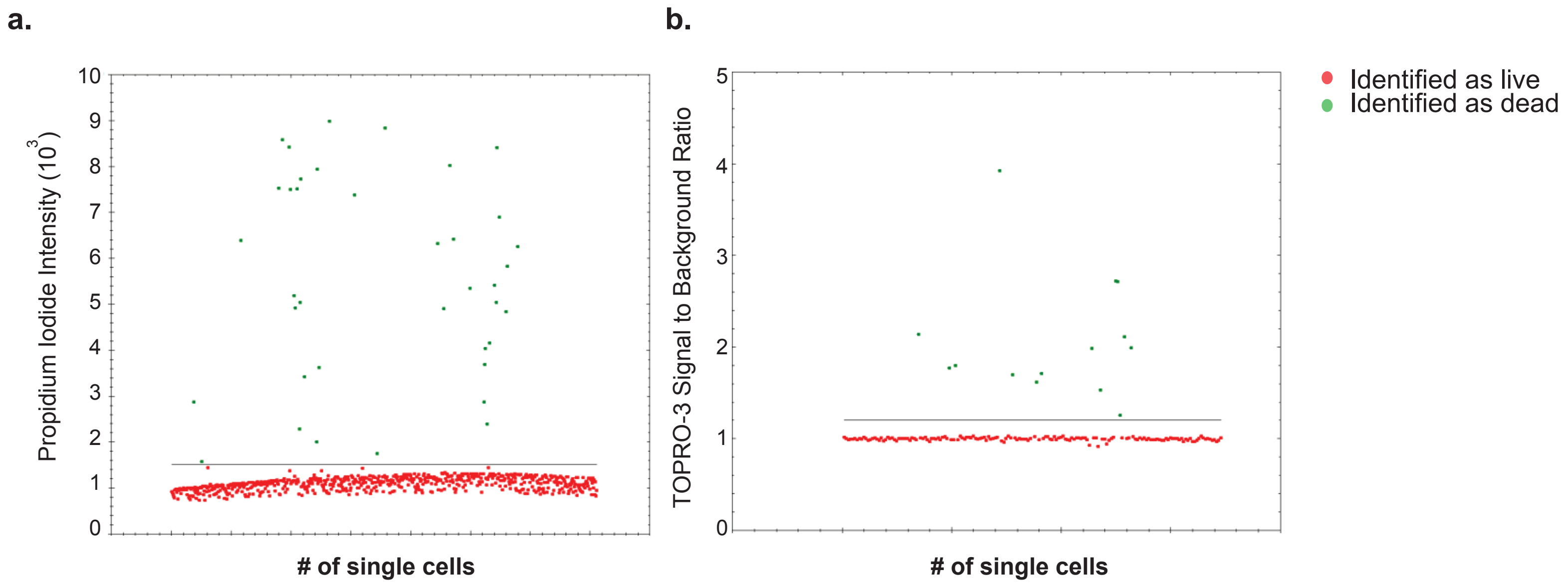

Supplementary Figure 2 - MTS assay fails to capture cytotoxic response of drug. H3255 and H3255R cells were treated with various concentrations of erlotinib and their viability was assessed after 72 hours via MTS assay. While we previously showed that erlotinib elicited increased cell death in H3255 cells with our HCS system, we are not able to detect this with MTS and only see a decrease in overall percent viability.

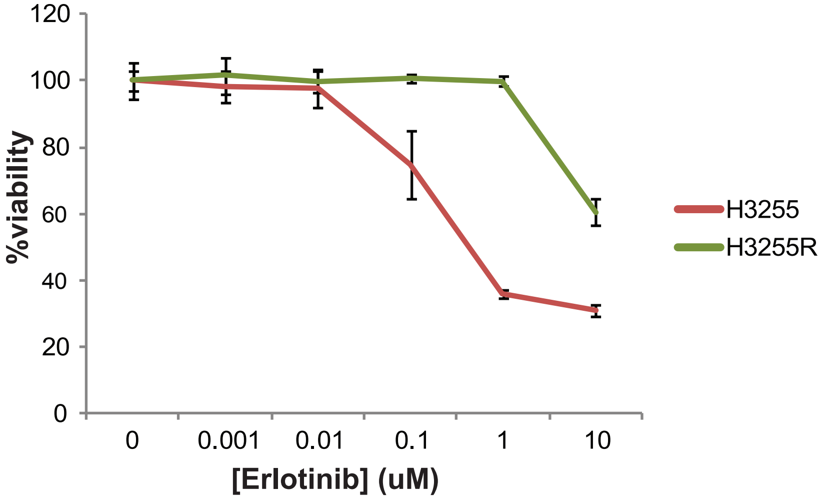

Supplementary Figure 3 - Initial Seeding density affects cellular response to drug. HCC827 cells were seeded at the desired density and treated with erlotinib. Their overall response is highly dependent on the initial seeding density, which is often not taken into account with traditional assays.

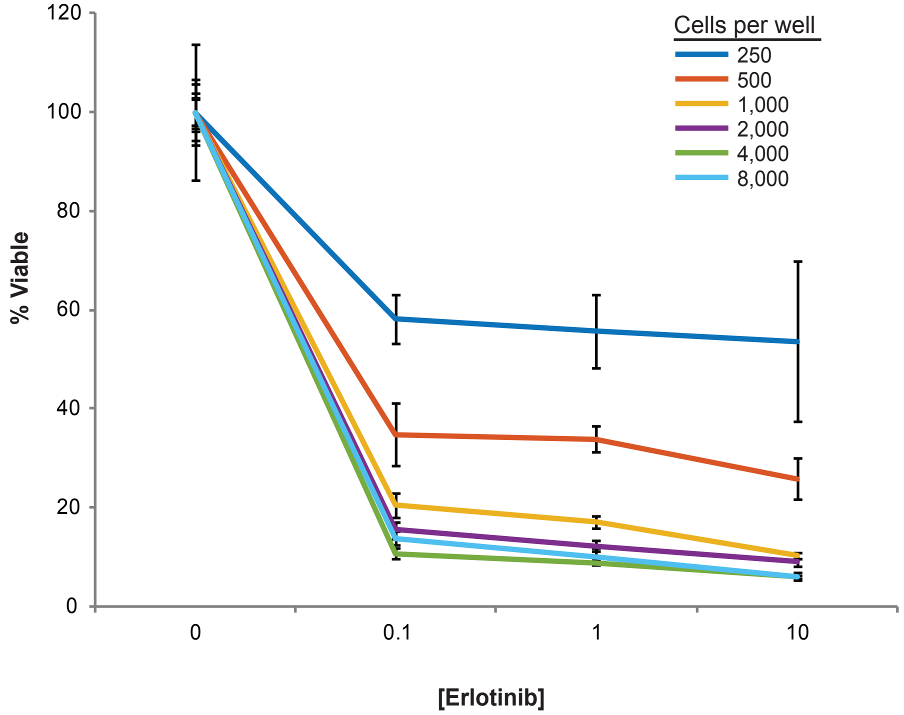

Supplementary Figure 4 - Birth and death rates of erlotinib resistant cell lines. Erlotinib resistant cell lines were assayed in conjunction with their isogenic parental lines (Fig. 2a), an advantage to thehigh-throughput capability of the platform, and their birth and death rates were calculated as a function of erlotinib and oxygen concentrations. Growth was slowed in both cell lines in response to decrease in oxygen and the highest level of erlotinib.

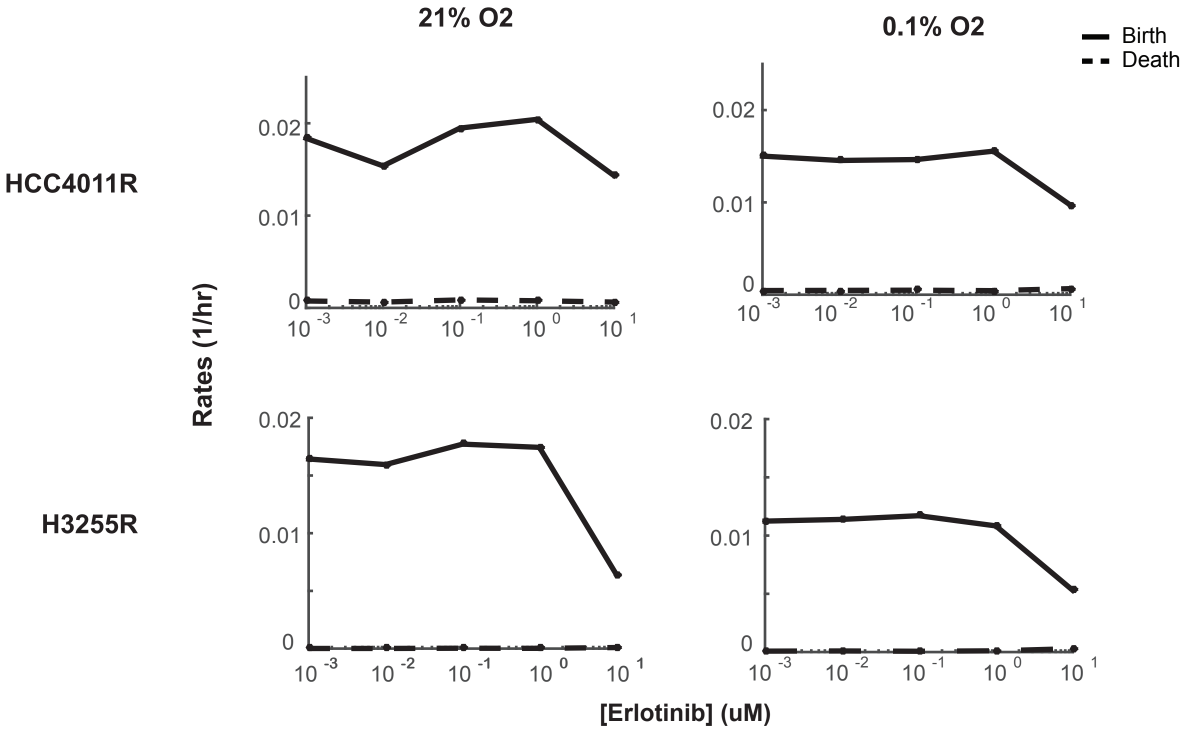

Supplementary Figure 5 - Comparison of morphology quantitation across software platforms. (a) Nuclear area distributions were tracked over time for H3255 cells in the presence and absence of erlotinib. CellProfiler and Harmony downstream analyses was performed on the same dataset, and the comparison is shown. (b) Cell area distributions in the presence and absense of 72 hour erlotinib incubation were calculated by both CellProfiler and Harmony. (c) Representative images of CellProfiler nuclear segmentation of H3255.

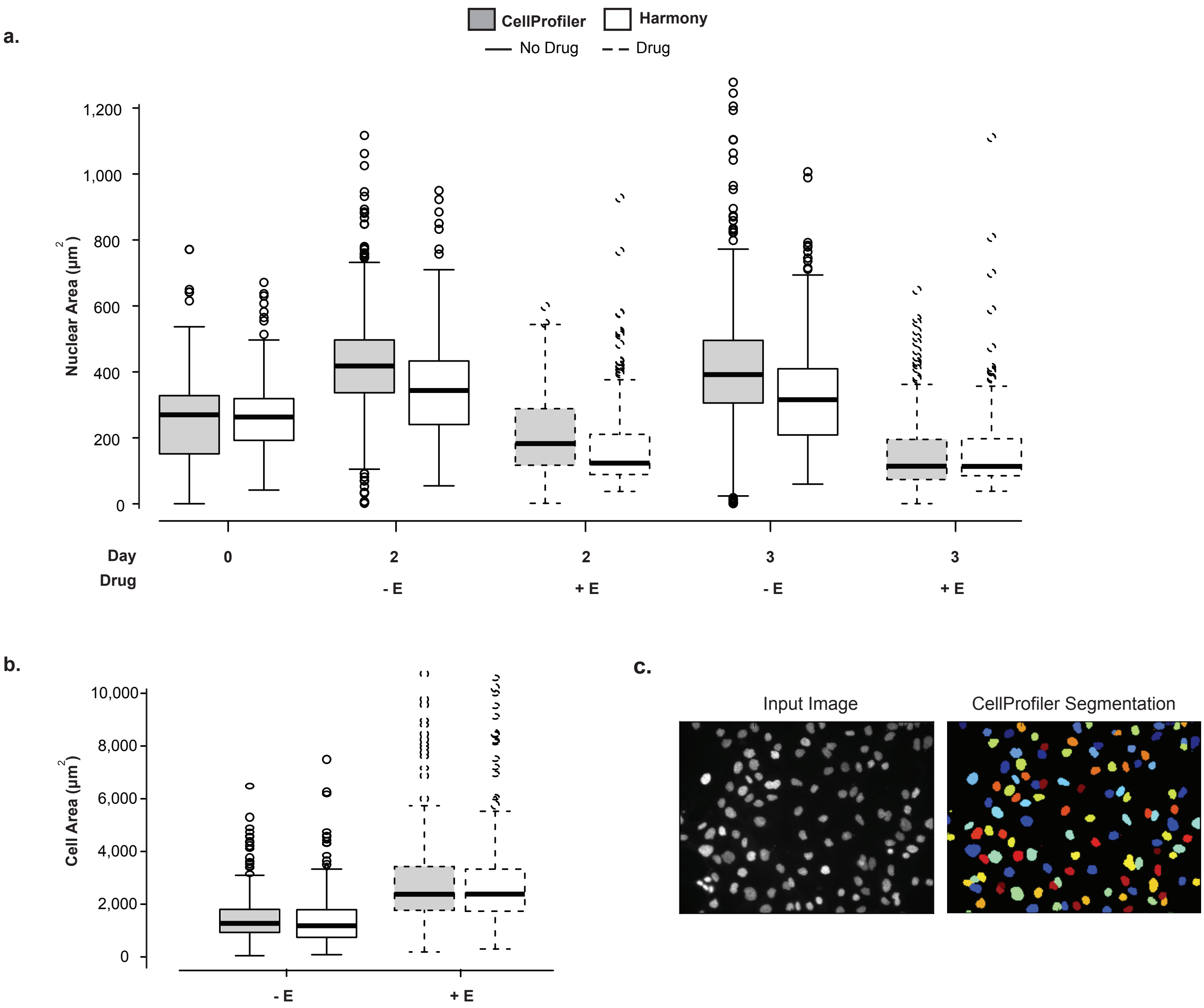

Supplementary Figure 6 - Tracking population composition over time and treament. H3255 and CCD-19Lu (lung fibroblast) cells were co-cultured at a 4:1 ratio and cell types were classified based upon morphological characteristics. Tumor composition (live and dead H3255 vs CCD-19Lu cells) was calculated over time in response to erlotinib (1 μM) and oxygen perturbation (21% vs 0.1%).

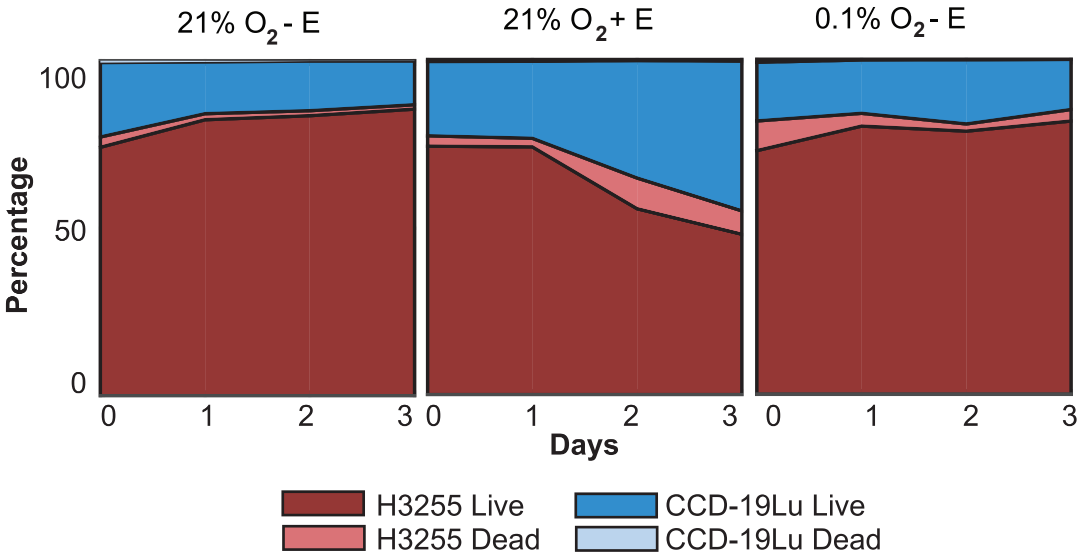

Supplementary Figure 7 - Mathematical model predictions. The mathematical model was parameterized using birth and death rates of H3255 sensitive and resistant cell lines at 21% oxygen concentration measured under 0.1 μM and 0.01 μM erlotinib. For each drug concentration, model predictions of the total tumor size (black lines), sensitive cell population (red lines), and resistant cell population (green lines) are plotted. An initial population of 4,000 cells was considered, with 1% pre-existing resistance and a mutation rate of 10^{-7}. (Note: solid green and solid black lines are overlapping.)

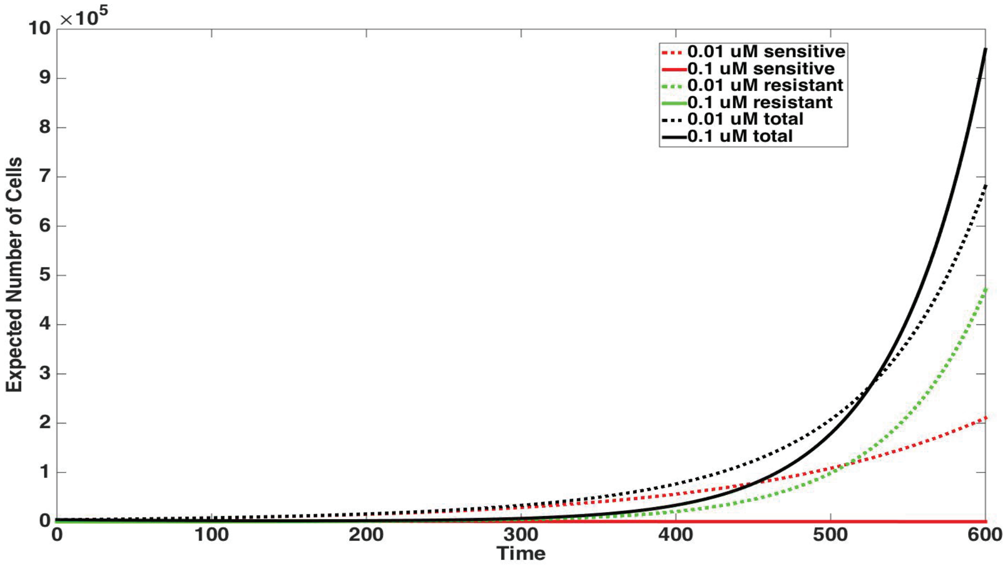

Supplementary Figure 8 - Live cell imaging across days. PC9 cells expressing histone-2B-GFP were imaged nine times over seven days (every 12 or 24 hours). Nuclei were segmented based off of GFP intensity and live cell analysis was performed on the same cells, with representative images from a single field of GFP-labeled nuclei over time shown.

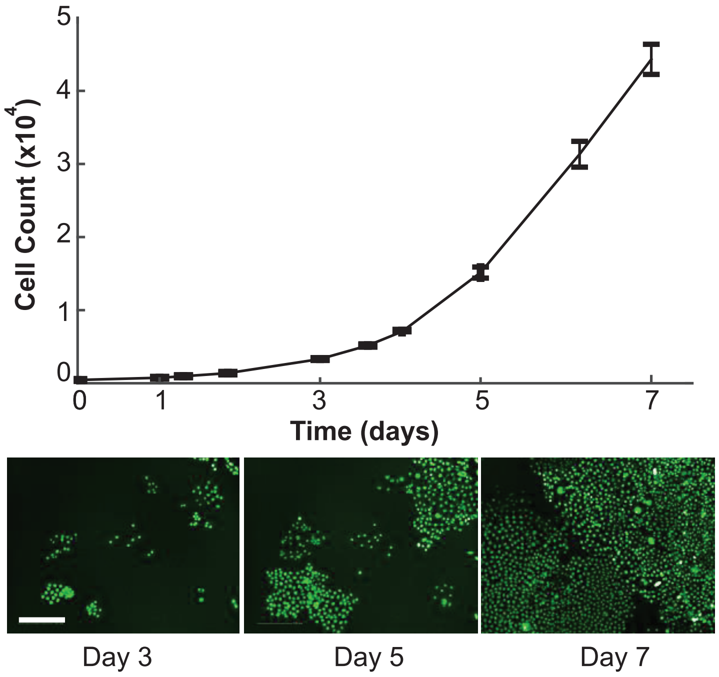

Supplement: Supplementary Information [file srep29752-s1.pdf]
